# APC/C^CDC20^ and APC/C play pivotal roles in the process of embryonic development in *Artemia sinica*

**DOI:** 10.1038/srep39047

**Published:** 2016-12-19

**Authors:** Mengchen Zhang, Feng Yao, Hong Luan, Wei Zhao, Ting Jing, Shuang Zhang, Lin Hou, Xiangyang Zou

**Affiliations:** 1 College of Life Sciences, Liaoning Normal University, Dalian 116081, China; 2 Department of Biology, Dalian Medical University, Dalian 116044, China

## Abstract

Anaphase Promoting Complex or Cyclosome (APC/C) is a representative E3 ubiquitin ligase, triggering the transition of metaphase to anaphase by regulating degradation and ensures the exit from mitosis. Cell division cycle 20 (CDC20) and Cell division cycle 20 related protein 1 (CDH1), as co-activators of APC/C, play significant roles in the spindle assembly checkpoint, guiding ubiquitin-mediated degradation, together with CDC23. During the embryonic development of the brine shrimp, *Artemia sinica*, CDC20, CDH1 and CDC23 participate in cell cycle regulation, but the specific mechanisms of their activities remain unknown. Herein, the full-length cDNAs of *cdc20* and *cdc23* from *A. sinica* were cloned. Real-time PCR analyzed the expression levels of *As-cdc20* and *As-cdc23*. The locations of CDH1, CDC20 and CDC23 showed no tissue or organ specificity. Furthermore, western blotting showed that the levels of *As*-CDC20, securin, cyclin B, CDK1, CDH1, CDC14B, CDC23 and geminin proteins conformed to their complicated degradation relationships during different embryo stages. Our research revealed that *As*-CDC20, *As*-CDH1 and APC mediate the mitotic progression, downstream proteins degradation and cellular differentiation in the process of embryonic development in *A. sinica*.

*Artemia sinica*, a small crustacean termed the brine shrimp, is widely distributed in hypersaline environments in China and is an important natural food source (the nauplii contain abundant proteins and unsaturated fatty acids). *A. sinica* has convenient breeding characteristics; therefore, it widely used in aquaculture and experimental studies^1^,^2^,^3^. *Artemia* have a strong ability to resist stresses, such as high salt, dryness, the absence of oxygen, cold, lack of food, and other severe conditions. To achieve this, they produce dormant cysts that are arrested at the gastrulation stage during embryonic development. This unusual diapause process is crucial to resisting poor environments during *Artemia* embryo development. Therefore, the causes and molecular mechanisms of diapause termination, and further the regulation of the cell cycle in *Artemia* embryos, have becoming a hot topic.

In mitosis, cell proliferation follows a complex, but orderly cell cycle, depending upon a number of essential cell-cycle regulatory proteins, such as cyclins and kinases. The ubiquitination-proteasome pathway mediates proteolysis, which modulates a series of cellular processes, such as apoptosis, cellular differentiation, chromosome segregation, cytokinesis, protein activation and degradation^4^,^5^. Ubiquitination through a variety of enzymes, (ubiquitin-activating enzyme, E1; ubiquitin-conjugating enzyme, E2; ubiquitin ligase, E3), degrades numerous specific regulatory cell-cycle substrates^6^. In mitotically dividing cells, the anaphase promoting complex or cyclosome (APC/C), a 1.5MDa multi-subunit ubiquitin ligase that regulates mitosis, participates in this specific proteolysis process^7^,^8^.

APC/C insures the accuracy of the cell cycle and the exit from mitosis, and activates the transition from metaphase to anaphase, by targeting specific mitotic regulators for proteolysis at distinct times during mitosis^9^,^10^. There are 19 different subunits assembled in APC/C, separated into four parts: tetratricopeptide repeat (TPR) subunits including cell division cycle 23 (CDC23); the catalytic core; the supporting structural composites; and co-activators: CDC20 and CDH1, which are used for substrate recognition^11^,^12^. APC/C activity is only detected in mitosis and G1 phase; however, the complex persists through the cycle, which is most likely related to the process of the embryonic development. The regulators of APC/C’s enzyme activities are CDH1 and CDC20, the choice of which depends mainly on the stage of the cell cycle^13^,^14^.

CDC20 and CDH1 associate with APC/C at different stages of the cell cycle, with the help of CDC23, facilitate substrate hydrolysis^15^,^16^. CDC20 is activated at the metaphase-anaphase transition via bonding with highly phosphorylated APC/C, peaking before CDH1’s dephosphorylation^15^,^17^. In other words, CDH1 reacts until the end of mitosis but continues into the G1 phase. CDC20 turns into APC/C^CDC20^ and participates in a mechanism called spindle assembly checkpoint (SAC), which mediates the separation of sister chromatids and ensures the correct orientation of chromatids^18^,^19^. SAC reduces the activity of APC/C^CDC20^ by preventing separase from being dephosphorylated^20^. The two key anaphase inhibitors, cyclin B and securin, were degraded at the metaphase-anaphase transition^21^, which results in the release of separase and the disassociation of the sister chromatids via cleaving cohesin. Following cyclin degradation, the activity of cyclin-dependent kinase 1 (CDK1) is reduced, permitting CDC14 to promote reactivated CDH1 to associate with APC/C^22^,^23^,^24^. In late anaphase, APC/C^CDH1^, rather than APC/C^CDC20^, performs protein ubiquitination, such as geminin, an inhibitory protein that prevents abnormal DNA replication^25^. Furthermore, the APC/C complex largely supports the binding of CDC20 and CDH1, and inducing the unstable destruction of CDC20 during S phase and early mitosis^26^.

These complex functions of CDH1 and CDC20 depend on their conserved structures. Human CDC20 and CDH1 are characterized by an “IR tail” at the C terminus and a “C-box” motif in the N-terminal region that are necessary for combining with APC/C^27^,^28^. In addition, these two proteins have highly conserved sequences, which often comprise seven blades called the WD40 domain β propeller^29^. During the cell cycle, the ubiquitination and degradation of proteins rely on the presence of the destruction-box sequence^30^,^31^, which is widely found in cyclins and securin^32^. D-box is a conserved region recognized by CDH1 and CDC20; another special sequence is the KEN box, which is present in CDC20 and geminin, and can be recognized by CDC20 and CDH1^33^.

Previous studies identified *cdc20* and *cdc23* in a variety of species, including yeast, invertebrates, vertebrates, mammals and humans. *Cdc20* encodes an important activator; and CDC23 is a core subunit of APC/C, which are essential for embryonic viability^34^. Their corresponding orthologs in mammals are also essential for completion of the cell division cycle^35^. However, the expression pattern, distribution and roles of *cd*c20, *cdc23* and CDH1 in early embryo development in *A. sinica* still remain unknown.

In the present study, to determine whether CDC20 and APC subunits are involved in regulation of diapause embryo development in *A. sinica*, the full-length cDNAs of the *As-cdc20* and *As-cdc23* were cloned. Their mRNA and protein expression levels during embryonic development were investigated using quantitative real-time PCR (qPCR) and western blotting. Whole mount immunohistochemistry (IHC) assayed the location of *As-*CDH1 expression. The location of *As-*CDC23 and *As-*CDC20 were tested by immunofluorescence (IF). We demonstrated the co-localization of CDH1 and CDC20 by double-staining immunofluorescence. Our aim was to further understand the function of CDC20, CDH1 and APC/C in the regulation of the cell cycle during early embryonic development in *A. sinica*.

## Results

### Cloning of *As-cdc20, As-cdc23* and bioinformatic analysis

An 1842 bp full-length cDNA of *As-cdc20* was obtained (GenBank accession: **KP162166**), which contained a 1512 bp ORF, a 50 bp 5′-UTR and a 280 bp 3′-UTR (Supplementary Fig. S1a). The putative *As*-CDC20 protein comprised 503 amino acids, and had a calculated molecular mass of 55.5 kDa and a pI of 8.97. SMART predicted a WD40 superfamily domain containing seven WD40 repeat sequences, which is a major feature of CDC20 proteins (Supplementary Fig. S1b). The PSORTII program predicted that CDC20 was 82.6% likely to be in the nucleus, 8.7% in the mitochondria, 4.3% in vesicles of the secretory system and 4.3% in plasma membrane. CDC20 is not a secretory protein: SignalP 4.0 did not predict a signal peptide in the protein. The protein sequence contained 30 predicted sites with a high probability (score 0.5) of being phosphorylated: 14 serines, 10 threonines and 6 tyrosines. The TMHMM Server 2.0 showed that *As*-CDC20 has no transmembrane helices, indicating that CDC20 is not a transmembrane protein. Protscale indicated that the putative protein was most likely to be hydrophilic (MIN: −2.978, MAX: 1.356).

Multiple sequence alignment analysis of the protein sequence of *As*-CDC20 revealed a highly conserved amino acid sequence between *A. sinica* and 17 other species from GenBank (Supplementary Fig. S2). CDC20 has a conserved WD40 domain, comprising a glycine-histidine (GH) dipeptide at the N-terminus; and a tryptophan-aspartic (WD) dipeptide of the C-terminus. To evaluate the evolutionary relationships of CDC20 proteins, we constructed a phylogenetic tree using the neighbor-joining method in Mega4.1. Protein sequences from 18 species were used to construct the phylogenetic tree (Supplementary Fig. S3), which showed five main clusters: mammal; vertebrates, which were separated as representative branches of the chordates and the echinodermatas; arthropods; and nematodes. The relationships displayed in the phylogenetic tree corresponded to their taxonomic classifications.

The full-length cDNA of *As-cdc23* was 1215 bp with APC8 domain in (Supplementary Fig. S4). The putative *As*-CDC23 protein contained 146 amino acids, a calculated molecular mass of 17.1 kDa and a pI of 5.95. SignalP 4.0 analysis predicted that *As*-CDC23 had no signal peptide. TMHMM Server 2.0 indicated that CDC23 is not a transmembrane protein. Protscale indicated that the putative protein was most likely to be hydrophilic. CDC23 subcellular localization was shown by PSORTII program predicted that it was 69.6% likely to be in the nucleus and 21.7% in mitochondria.

Bioinformatics analysis suggested that CDC23 had the highest sequence similarity with the same protein from the southeastern blueberry bee, *Habropoda laboriosa* (Supplementary Fig. S5). The CDC23 sequences of 16 species were selected to construct a neighbor-joining phylogenetic tree (Bootstrapping = 1000) (Supplementary Fig. S6), in which species were classified in three clusters: vertebrates (contains mammals), arthropods and nematodes, with *As*-CDC23 belongs to the arthropods. The relationships displayed in the phylogenetic tree corresponded to their taxonomic classifications.

### Expression pattern of *As-cdc20* and *As-cdc23* in different developing stages

**Figure 1:**
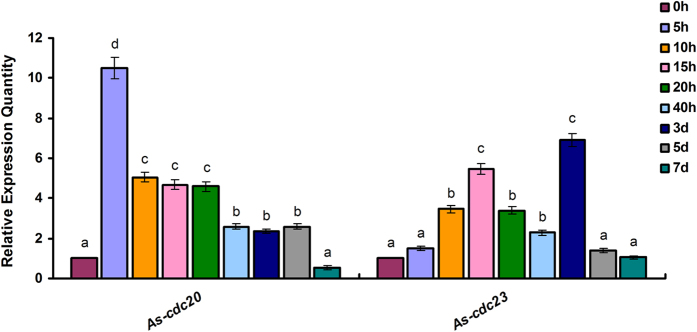
Real-time qPCR analysis of *As-cdc20* and *As-cdc23* expression levels at different developmental stages of *A. sinica*. Significant differences at different development stages (*P* < 0.05) were analyzed by multi-factor analysis of variance (MANOVA) and indicated by lowercase letters (a, b and c).

To determine the amount of *As-cdc20* transcription during the development of *A. sinica*, qPCR analysis was performed (Fig. 1). The results showed that the amount of *As-cdc20* mRNA began to increase rapidly during the developmental stages from 0 h to 5 h, reaching a notably high level at 5 h. Thereafter, the transcript level decreased from 10 h to 20 h, and decreased remarkably from 3 to 5 d, until at 7 d, the expression was at a very low level. The expression analysis of *As-cdc23* also shown in Fig. 1: the transcript level increased from 0 h to 10 h, decreased until 40 h, suddenly peaked at 3 d and thereafter expressed at a low level.

### Expression and purification of *As-*CDC20 and *As*-CDC23

**Figure 2:**
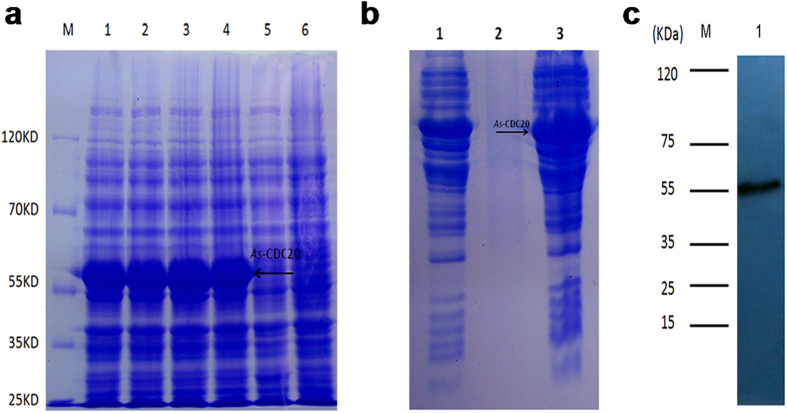
The results of prokaryotic expression and antibody detection of *As*-CDC20 protein. **(a)** Expression analysis of the recombinant *As*-CDC20 protein. **(b)** Detection of the solubility of the recombinant *As*-CDC20 protein. **(c)** Western blotting analysis of the specific binding of the purified recombinant protein with antiserum antibodies. The full-length gels are presented in Supplementary Fig. 7.

In Fig. 2a, Lanes 1–4 show the expression of the recombinant *As*-CDC20 protein from four induction treatments (1 mM IPTG at 37 °C; 1 mM IPTG at 30 °C; 0.25 mM IPTG at 37 °C and 0.25 mM IPTG at 30 °C, respectively), and there was no significant difference between them. Lane 5 shows total proteins from non-induced cells; Lane 6 shows total proteins from induced cells harboring pET-30a (control). Therefore, a predicted 55.5 kDa protein of *As-*CDC20 was successfully expressed in *E. coli* using the pET30a vector; the observed molecular mass of the recombinant protein was about 55 kDa, which agreed with the prediction. We chose 0.25 mM IPTG at 30 °C for further experiments. SDS-PAGE showed in Fig. 2b: Lane 1: total pET-30a-CDC20 recombinant protein; Lane 2: soluble fraction of the lysate from induced cells harboring pET-30a-CDC20; Lane 3: insoluble fraction of the lysate from induced cells harboring pET-30a-CDC20, in which the recombinant protein existed. Western blotting was performed to verify that the preparative antibody could bind specifically to the purified protein (Fig. 2c).

**Figure 3:**
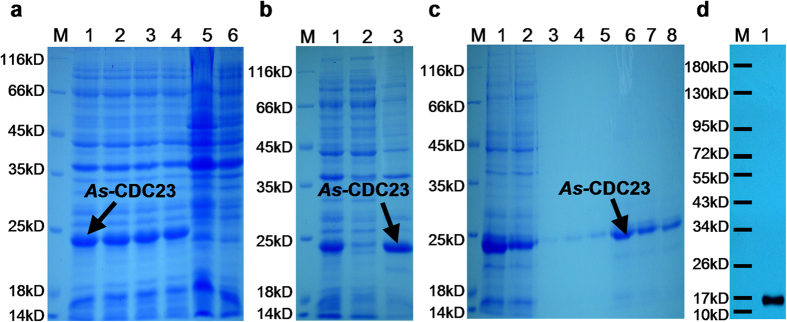
The results of prokaryotic expression and antibody detection of *As*-CDC23 protein. **(a)** Expression of the recombinant *As*-CDC23 protein. **(b)** Detection of the solubility of the recombinant *As*-CDC23 protein. **(c)** Purification of the recombinant *As*-CDC23 protein. **(d)** Western blotting analysis of the specific binding of the purified recombinant protein with antiserum antibodies. The full-length gels are presented in Supplementary Fig. 8.

The *As-*CDC23 protein was expressed recombinantly via the pET28a vector. SDS-PAGE analysis showed the recombinant protein in Fig. 3a: The orders of Lanes are same as Fig. 2a, but Lane 6 shows the total proteins from induced cells harboring pET-28a (control). The recombinant protein was approximately 17 kDa; and 1 mM IPTG at 37 °C was chosen for further experiments. SDS-PAGE showed that the recombinant protein existed in the insoluble fraction of the lysate from induced cells harboring pET-28a-CDC23 (Fig. 3b, Lanes order refers to Fig. 2b). The purification analysis of *As*-CDC23 shown in Fig. 3c: Lane 1: total proteins extracted from induced cells harboring pET-28a-CDC23. Lane 2 shows flow through eluate of total proteins. Lanes 3–8: column elution with eluant containing 10 mM, 20 mM, 40 mM, 60 mM, 80 mM and 100 mM imidazole, respectively. Therefore after denaturation in 80 mM urea, followed by purification and dialysis, a relatively pure protein was obtained. Western blotting verified that the preparative antibody could bind specifically to the purified protein (Fig. 3d).

### Protein expression patterns of interacting proteins in different developing stages

**Figure 4:**
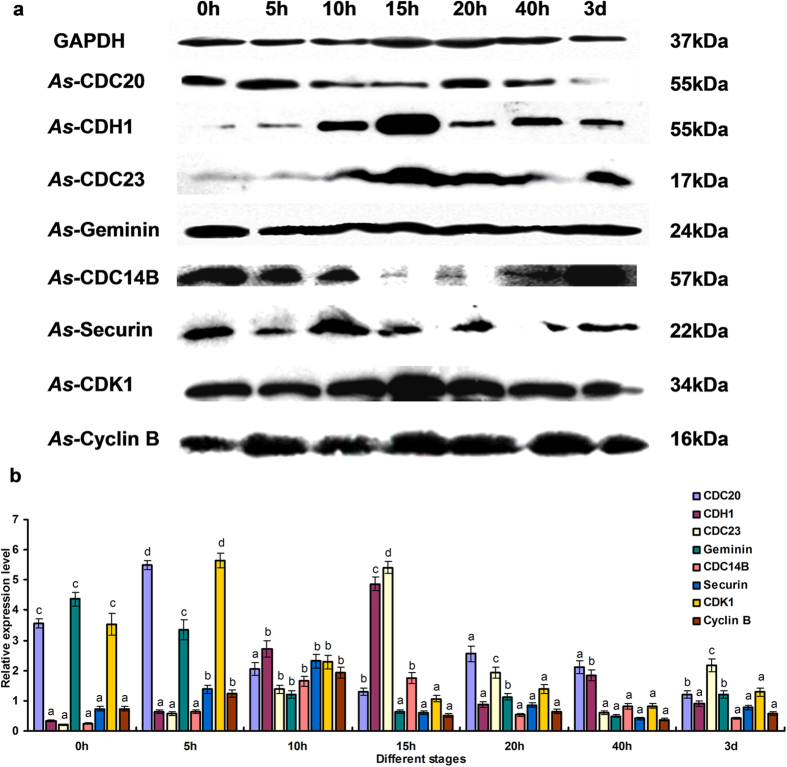
Western blotting analysis of *As*-CDC20, *As*-CDH1, *As*-CDC23, *As*-CDC14B, *As*-Securin, *As*-Cyclin B, *As*-CDK1 and *As*-Geminin. **(a)** Western blotting showing the expression of proteins at different developmental stages of *Artemia sinica*. **(b)** Values are expressed as arbitrary units of relative value. Significant differences (*P* < 0.05) were analyzed by MANOVA and indicated by lowercase letters (a,b,c and d). The images are cropped, and the full-length blots are presented in Supplementary Fig. 9.

The intensities of the proteins bands were normalized against those of GAPDH. The expression level of *As*-CDC20 at different developmental stages showed a rising trend from 0 h to 5 h, at which point it reached its highest level. Thereafter, it showed a downward trend at 10 h and remained relatively stable during the following stage. The expression level reached its lowest at the metanauplius stage 3d (Fig. 4). The expression of *As*-CDC23 and CDH1 revealed a distinct upward trend from 0 h to 15 h, followed by a downward at 20 h, and then remained stable to 3 d. CDC14B, cyclin B and securin expression levels were similar to CDH1, except for a peak at 10 h. Moreover, the expression of CDK1 and geminin showed the opposite expression mode to CDH1.

### Localization of *As*-CDH1 expression by immunohistochemistry

**Figure 5:**
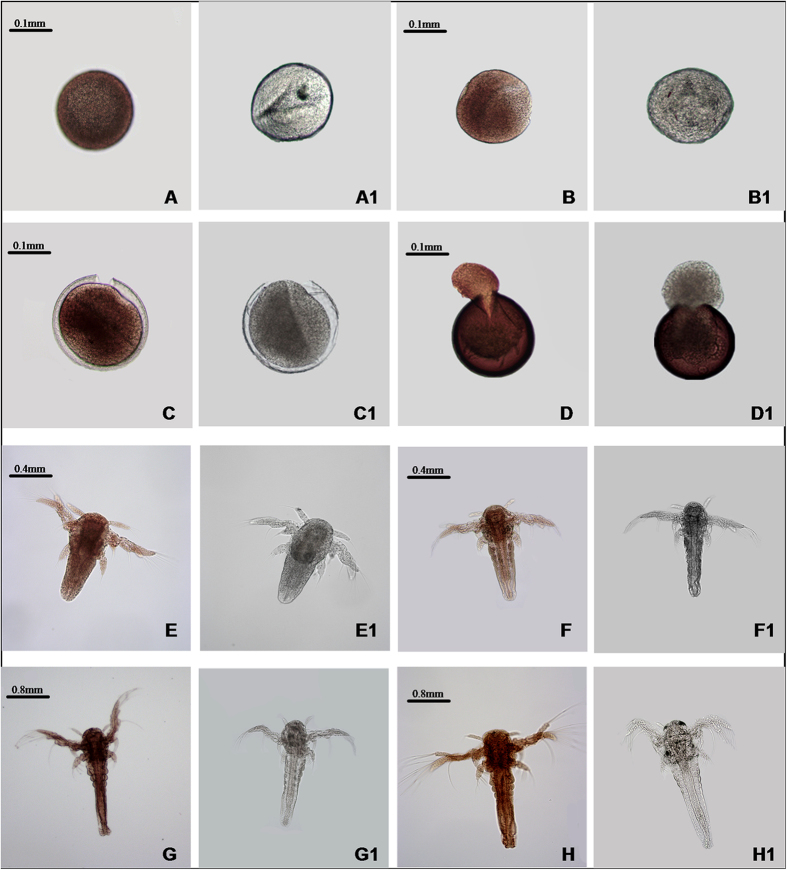
The whole mount immunohistochemical analysis of *As*-CDH1 expression during different developmental stages in *A. sinica*. (**A–H**) represent the experimental groups; A1–H1 represent the control group. (**A**) gastrula stage (0 h); (**B,C,D**) embryonic stage (5 h, 10 h and 15 h); (**E,F**) nauplius stage (20 h and 40 h); (**G,H**) metanauplius stage (3 and 7 days).

Whole mount immunohistochemistry of CDH1 showed low expression at 0 h (gastrula stage; Fig. 5A), thereafter the expression showed an increasing tendency from 5 h to 10 h (Fig. 5B,C) throughout the cyst. At the umbrella stage (15 h), the cyst head broke through the shells with the tail remaining inside (Fig. 5D), at which point *As*-CDH1 expression was very high. From the 20 h stage, CDH1 was detected throughout the whole body (Fig. 5E), especially after 40 h, expression focused in cephalothorax and abdomen (Fig. 5F,G and H). The negative control (incubated with 1 × PBS) showed no positive signals (Fig. 5A1–H1).

### Immunofluorescence analysis of *As*-CDC20, CDC23 and CDH1

**Figure 6:**
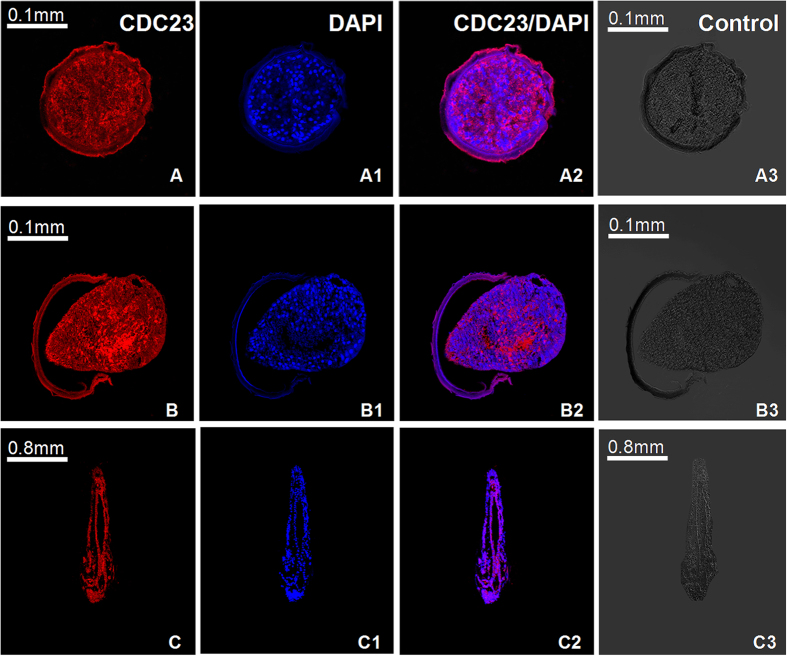
Immunolocalization analysis of *As*-CDC23 at the embryo and sub-adult stages in *A. sinica*. A-A3: 0 h; B-B3: 15 h; C-C3: 3d; (**A–C**) represent anti-CDC23-label; A1-C1: single-labeling with DAPI; A2-C2 represent the image overlay of the samples dual-labeled with polyclonal anti-CDC23 and DAPI. A3-C3 represent control group.

To observe the localization of *As*-CDC23 and double-labeled IF of CDH1 and CDC20, we used a laser scanning confocal microscope (LSCM) to observe the embryos (0 h, 15 h) and metanauplius larvae (3 d). In the *As*-CDC23 experimental groups, the *As*-CDC23 reactivity was observed throughout the embryo (Fig. 6A,B) and the whole body of the sub-adult stage (Fig. 6C). The cell nuclei of *A. sinica*, labeled by DAPI, were detected in the embryo (Fig. 6A1,B1) and sub-adult (Fig. 6C1). The merged images of the two fluorescences in the embryo (Fig. 6A2,B2) and sub-adult stage (Fig. 6C2) showed that the distribution of *As*-CDC23 mostly was coincident with the cell nucleus. Control groups incubated with 1×PBS showed no signal above the background (Fig. 6A3,B3,C3).

**Figure 7:**
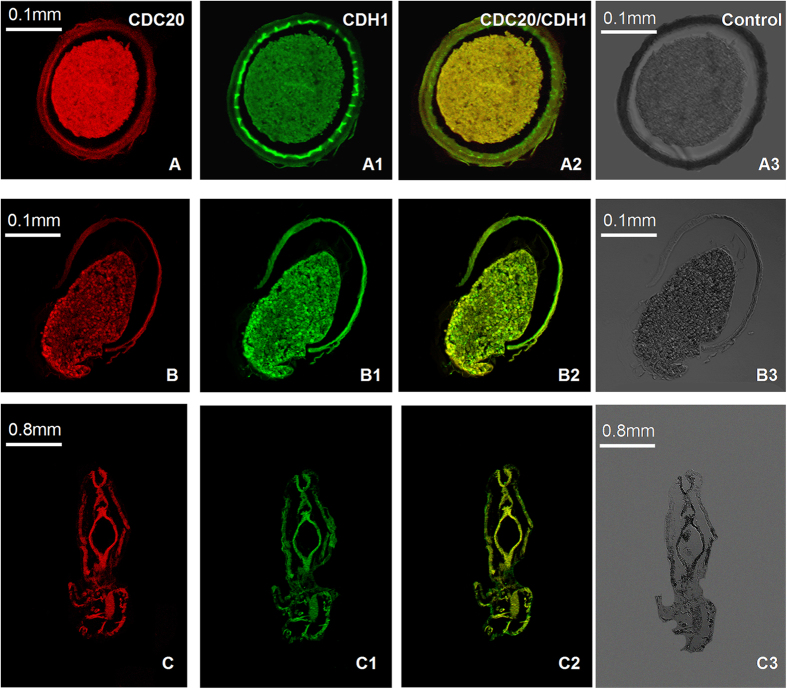
The double-labeled immunofluorescence analysis of *As*-CDC20 and *As*-CDH1 in *A. sinica*. A–A3: gastrula stage; B-B3: embryonic stage; C-C3: metanauplius stage; (**A,B** and **C**) represent single-labeling with anti-CDC20; A1-C1: single-labeling with anti-CDH1; A2-C2 represent the image overlay of dual-labeled samples. A3–C3 represent the control group.

In the double-labeled IF assay of *As*-CDC20 and *As*-CDH1, CDH1 was observed to be expressed throughout the embryo (Fig. 7A,B) and metanauplius (Fig. 7C), similar to CDC20 in the embryo (Fig. 7A1,B1) and metanauplius (Fig. 7C1). The merged images of the two fluorescences in the embryo (Fig. 7A2,B2) and the sub-adult stage (Fig. 7C2) showed that the expression distribution of *As*-CDC20 could be nearly superposed on that for CDH1. Control groups showed no signal above the background (Fig. 7A3,B3 and C3).

## Discussion

WD40 super family contains a GH dipeptide and a WD dipeptide, between which is a conserved 40-amino-acid sequence; the sequences of WD-repeat proteins are conserved from humans, mammals to invertebrates^36^,^37^. These family proteins participate in signal transduction, the regulation of transcription, apoptosis and cell cycle control^38^,^39^. The *As-cdc20* cDNA encoded a putative protein of 503 amino acids, which includes a characteristic WD40 domain that highly conserved with other species, such as vertebrates, mammals and arthropod groups. The C-terminal WD40 domain included a KEN-box that causes the degradation by CDH1, and a D-box-binding site, which is critical for the ubiquitination of interacting proteins by APC/C^CDC20^ 32,40. *As*-CDC20 has no signal peptide or transmembrane regions, and is mostly localized in the nucleus, which is related to its role: an activator of APC/C, promoting cell cycle and the process of embryonic development^11^.

APC^CDC20^ plays pivotal roles in governing mitotic progression, especially in cancer cells as a carcinogenic factor. The detection of *cdc20* mRNA by qPCR in gastric cancer tissues showed that the mean expression value of *cdc20* mRNA in cancer tissues was significantly higher than in normal tissues. For example, 51.9% of gastric cancer tissues showed high CDC20 expression, but only 18.3% showed high expression in the adjacent noncancerous tissues. This implied that inhibiting the expression of *cdc20* could slow down the growth of tumor cells and induce cell stagnation at the G2/M phase^41^,^42^. From the qPCR and western blotting results, *As-*CDC20 existed in the gastrula stage as a maternal protein, and at 5 h, *A. sinica* broke the diapause status and cells began to divide rapidly; the amounts of *As-cdc20* transcripts were suddenly upregulated. This suggested that CDC20 might operate in a cell-cycle-dependent manner. By contrast, APC and CDH1 target CDC20 for destruction^19^, and both of them showed an upward expression trend during the developmental stages from 10 to 20 h, during which *As-cdc20* levels began to decrease. With the slow rate of cell differentiation after 40 h, the *cdc20* transcripts and CDC20 were maintained at a low level; associated with the high level of CDH1 and CDC23.

*As*-CDC23 is one of the TPR subunits, has a conserved APC8 domain in the N-terminus, similar to that in other species. *As-cdc23* transcripts increased when the hatching conditions were suitable for diapause termination, during which time embryonic cells are dividing actively and the synthesis of proteins becomes necessary; and downregulated when the larvae exposed to the external environment challenges. The TPR subunits behave as versatile recognition sites for several interacting proteins; take a helping part in the instability of CDC20 by APC/C^CDH1^ during G1 phase and the degradation of CDC20 during S phase and early mitosis^43^.

Previous studies showed CDC20, CDH1 and CDC23 exist widely among eukaryotes, from mammals to *Saccharomyces cerevisiae*^44^,^45^. Fluorescently labeled antibodies showed that *As*-CDC23 was distributed extensively throughout all parts, but particularly in the nucleus, according with its role: scaffold protein. The IF results showed that *As*-CDC20 was expressed in almost all parts of *A. sinica* during different developmental stages, which similar to the localization results of *As*-CDH1. Double-labeled immunofluorescence of CDH1and CDC20 showed that the two proteins were expressed in mostly the same place, in other words CDC20 and CDH1 combine with APC/C as nuclear co-activators in the cell cycle, to ensure that exit from mitosis does not occur before sister chromatids separation has been initiated^46^.

CDC20 is overexpressed in oral squamous cell carcinoma, colon cancer cell lines and primary cancer tissues, compared with normal noncancerous tissue samples. This suggested that CDC20 is associated with the progression of carcinoma^47^,^48^. As a promoter of cell proliferation function, CDCD20 participates in regulation of the cell cycle with APC/C. CDC20 also enriched at the centrosome in neurons, which is critical for CDC20-dependent dendrite development, and for the proliferation and differentiation of neural stem cells. CDH1 is an inhibitor, being downregulated in cancer cells, but is highly expressed in a variety of tissues that are predominantly composed of differentiated cells, such as the adult brain and the nuclei of terminally differentiated neurons^49^. APC^CDH1^ is essential for endoreduplication, and ablation of CDH1 might trigger replicative stress, cell cycle arrest, cell death, and even causing a hypoplastic brain and hydrocephalus in the developing nervous system^50^.

**Figure 8:**
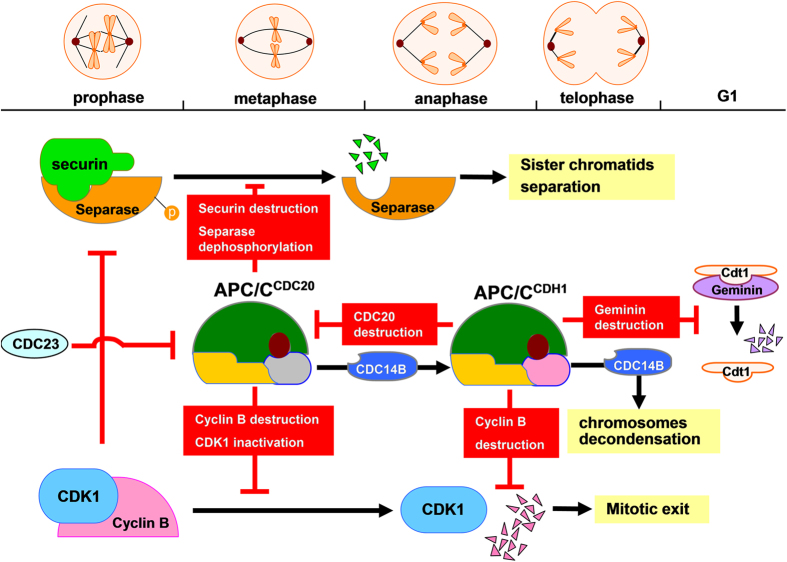
The separation of sister-chromatids and ubiquitin degradation by APC/C^CDC20^ and APC/C^CDH1^ in mitosis. Mitotic progression is stimulated by the destruction of securin, triggered by APC/C^CDC20^; and cyclin B, which is initiated by APC/C^CDC20^ and APC/C^CDH1^. APC/C^CDH1^ replaces APC/C^CDC20^ with CDC23 and CDC14B take part in, and mediates the downstream ubiquitination such as cyclin B and geminin.

In the cell cycles, the progression to anaphase is triggered crucially by the mitotic kinase and the separation of sister chromatids. In this progression, CDH1 and CDC20 form activated complexes with APC/C to initiate ubiquitination of downstream proteins. CDC20 levels accumulated from the G2 phase, peaking at metaphase when it associates with APC/C, initiating the degradation of securin via the D-box (destruction box) sequence, resulting in activation of the metaphase-anaphase transition^29^,^31^. Securin is an inhibitor of separase, after the ubiquitination of securin by APC/C^CDC20^, separases are released. This process triggers the degradation of cohesion, and then chromatids were cleaved^51^,^52^. In *A. sinica*, the 0 h cysts were treated to break diapause and the rate of proliferation began to increase. The expression levels of *As-*CDC20 showed a notably rising trend from 0 h to 5 h; accordingly, the expressions of securin showed a lower level. Therefore our results are consistent with previous data indicating that APC/C^CDC20^ degraded securin in the proliferation process of diapause termination and embryonic development in *A. sinica*. At the same time, levels of cyclin B began to rise, which leaded to a high CDK1 activity, and prevented CDH1 from binding with APC/C. By contrast, cyclin B destruction via APC/C^CDC20^ occurred at the end of anaphase, causing a reduction in CDK1 activity and an upregulation of CDC14B, which is a CDK-counteracting phosphatase; this triggered the removal of mitotic phosphate groups from CDH1, allowing it to associate with APC/C, and to target CDC20 and other substrates for destruction^8^,^14^,^25^. From telophase, APC/C^CDH1^ replaced CDC20, working with CDC14B to promote decondensation of chromosomes, leading the exit from mitosis^52^,^53^. From the start of the S phase to late mitosis, geminin inhibits the replication factor Cdt1, preventing the assembly of the pre-replicative complex. In the G1 phase, CDH1 triggers the destruction of geminin, ensuring that only one round of replication occurs during each cell cycle^54^,^55^ (Fig. 8). Protein expression patterns studies indicated the levels of cyclin B, CDK1, CDH1, CDC14B and geminin proteins conformed to their complicated degradation relationships during different embryo stages of *A. sinica*. Thus, we hypothesized that the ubiquitination of downstream proteins by CDC20, CDH1 and APC/C might play decisive roles in early embryo and post-diapause development in *A. sinica*.

## Materials and Methods

### Animal preparation

No specific permits were required for our samples of *Artemia* cysts collected and field studies. The location was not privately owned or protected in any way, and the field studies also did not involve endangered or protected species. We confirm that the salt lake and land we conducted our study on was not privately owned or government protected.

*A. sinica* cysts were collected from Yuncheng salt lake (Shanxi, China)^56^, and kept at a low temperature to terminate diapause. The cysts were hatched in filtered seawater, at 28 °C, with a salinity level of 28‰ and a light intensity of 1000Lux, according to a previously described method^57^. Samples were collected at different times (0, 5, 10, 15, 20 and 40 h, and 3, 5, and 7 d) for subsequent experiments.

### cDNA cloning of *As-cdc20* and *As-cdc23*

Total RNA was extracted from 0 h cysts (hatched in axenic seawater for 30 min) using the Trizol Reagent Kit (Tiangen, Beijing, China), according to the manufacturer’s instructions. Reverse-transcription used an oligo(dT) primer and MLV reverse transcriptase (Takara, Dalian, China) to obtained cDNA. We designed primers using Primer Premier 5.0 to obtain expressed sequence tags (ESTs) representing *cdc20* and *cdc23*, which were obtained from *Artemia franciscana* sequence, in GenBank. All gene-specific primers used for the cloning of *As-cdc20* and *As-cdc23* were synthesized by Sangon (Shanghai, China) and are shown in Table 1. The PCR reactions were performed as follows: initial incubation at 94 °C for 5 min; followed by 30 cycles of amplification (denaturation at 94 °C for 30 s, annealing 30 s at 60 °C for *cdc20,* 52 °C for *cdc23* and elongation at 72 °C for 1 min) with a final incubation at 72 °C for 10 min. The PCR products were separated on 1.0% agarose/TAE gels, purified and cloned into the pEASY vector (TransGen, Beijing, China), then sequenced by Sangon. Thus, a 214 bp EST of *As-cdc20* and a 431 bp EST of *As-cdc23* were obtained.

The full-length sequence was obtained by 3′ and 5′ rapid amplification of cDNA ends (RACE) using a 3′- Full RACE Amplification Kit (Takara) and a 5′ SMART RACE cDNA Kit (Clontech, CA, USA), according to the supplier’s instructions; the RACE PCR primers are shown in Table 1. The target RACE products were purified and sequenced; two termination fragments were spliced together using DNAMAN to yield the full-length cDNA of *As-cdc23* (GenBank: **KU986660**) and *As-cdc20* (GenBank: **KP162166**).

### Bioinformatics analysis

The identities and similarities of *As-cdc20* and *As-cdc23* were analyzed by National Center for Biotechnology Information (NCBI) online Search Tool (BLASTX) (http://blast.ncbi.nlm.nih.gov/Blast.cgi) and the open reading frames (ORFs) were identified using ORF Finder (http://www.ncbi.nlm.nih.gov/gorf/gorf.html). The prosite tools of ExPASy (http://prosite.expasy.org/prosite.html/) and SMART (http://smart.embl-heidelberg.de/) predict the protein structural and functional domains. The ProtParam tool of ExPASy (http://web.expasy.org/protparam/) also predicts the molecular weight and theoretical isoelectric point (PI) of the proteins. Psort II (http://psort.hgc.jp/form2.html) was used to predict the subcellular localization. SignalP 4.1 (http://www.cbs.dtu.dk/services/SignalP/) can predict signal peptides. TMHMM Server 2.0 (http://www.cbs.dtu.dk/services/TMHMM-2.0/) and Protscale (http://web.expasy.org/protscale/) were used to detect transmembrane helices and to calculate hydrophobicity and hydrophilicity, respectively. Subsequently, multiple sequence alignments were performed for the amino acid sequences of *As-*CDC20 and *As-*CDC23 with similar proteins from other species, using the ClustalX2.0 and DNAMAN. MEGA4.1 software and ClustalX2.0 were used to construct a phylogenetic tree of the two proteins from different species using the neighbor-joining (NJ) method. The statistical significance of groups within the phylogenetic trees was evaluated using the bootstrap method with 1000 replications.

### Quantitative real-time PCR (qPCR)

RNA samples of *A. sinica* were extracted from different growth periods (0–7 d) and cDNA templates were prepared using the method detailed in Section 2.2. QPCR primers are shown in Table 1. Real-time PCR was performed in triplicate for every sample in a parallel design, using the SYBR Premix Ex Taq (Takara) and the Takara detection system, according to the manufacturer’s protocol. PCR reaction conditions according to the previously described method^56^,^57^. The *A. sinica β-*actin gene was used as a normalization control for each starting quantity of RNA. Data were analyzed using the comparative cycle threshold (Ct) method (2^−ΔΔ^Ct method), based on Ct values for *As-cdc20, As-cdc23* and *β*-actin, to calculate the fold increase. Data obtained from qPCR analysis were analyzed by SPSS 16.0 software and the significance threshold of the means values between the treatment and control groups were set at *P* < 0.05.

### Expression and purification of recombinant *As*-CDC20 and *As*-CDC23 proteins

The complete ORFs were amplified using primers (Table 1) with specific restriction endonuclease recognition sites. The *cdc23* was cloned into vector pET28a and *cdc20* was cloned into vector pET30a. The recombinant plasmids and vectors were digested with the respective enzymes (BamHI & XhoI for *cdc20*; EcoRI & BamHI for *cdc23*) and then ligated together using T4 DNA (Takara) ligase at 16 °C overnight. The resulting expression vectors, pET-30a-*cdc20* and pET-28a-*cdc23*, were transformed into *Escherichia coli* BL21 (DE3) and cultured overnight at 37 °C. The expression of the fusion protein was induced by four different conditions: 1 mM IPTG for 3 h at 37 °C, 1 mM IPTG for 3 h at 30 °C, 0.25 mM IPTG for 3 h at 37 °C and 0.25 mM IPTG for 3 h at 30 °C. Cells were collected by centrifugation and ultrasonication. Purification of recombinant proteins was accomplished using HisTrap^TM^ FF crude (GE Healthcare, Beijing, China), following the supplier’s protocol. The imidazole concentrations of the elution buffer were: 10 mM, 20 mM, 40 mM, 60 mM, 80 mM and 100 mM. The protein was dialyzed into 20 mM Tris-HCl and then freeze-dried using a lyophilizer (Millrock, NY, USA).

### Production of polyclonal antibodies

Polyclonal antibodies directed against the recombinant proteins were prepared in rabbits. Rabbits were immunized every two weeks by multipoint intradermal injections. For the first immunization, the purified protein (600 μg/ml) was emulsified in an equal volume of Freund’s complete adjuvant. For the three subsequent immunizations, 300 μg/ml purified protein was emulsified with an equal volume of Freund’s incomplete adjuvant. The antiserum was collected from blood samples by centrifugation at 10000 g for 10 min, and the protein concentration was checked by an enzyme-linked immunosorbent assay (ELISA). The specificity of the antibody for the purified protein was determined using western blotting.

### Western blotting

Cyst samples were collected from different developmental stages (0–3 d). The total proteins were extracted from each sample using RIPA lysis buffer and quantified using the Bradford method^58^. One hundred micrograms of each sample were subjected to fractionation by SDS-PAGE and transferred to PVDF membranes. The membrane was blocked with 5% non-fat powdered milk (Sangon) and incubated with the primary antibodies at 4 °C overnight. Rabbit anti-*As*-CDC20 and anti-*As*-CDC23 polyclonal antibodies were diluted 1:200 with PBST; GAPDH antibody was diluted 1:1000. The membranes were incubated in HRP-conjugated goat anti-rabbit IgG (Proteintech, Wuhan, China; for CDC20 and CDC23); and anti-mouse IgG for GAPDH at 37 °C in 1 h. The reactive protein bands on the membrane were visualized using the ECL reagent (Beyotime, Shanghai, China) and exposed to X-ray film in the darkroom. The films were photographed and analyzed using Image J software, in which a gray scale of bands was used to compare the density of the immunoreactive bands, and the data was used to build column charts. The intensities of the specific proteins bands were normalized against those of GAPDH. Other antibodies such as CDC14B, geminin, CDH1, securin and CDK1 were bought from Proteintech and BOSTER (Wuhan, China); these antibodies were bought according to the sequence homology, of which all the homologies exceed 80%.

### Whole mount immunohistochemistry of *As*-CDH1

*A. sinica* cyst samples were collected at different developmental stages (0–5 d). Samples from 0, 5 h and 10 h were dechorionated using 20% NaOCl. All the samples were fixed using an equivalent volume of heptane and PEM-FA (contains PIPES, EGTA, MgSO_4_ and oxymethylene) for 20 min. The pellets were washed with methanol three times and stored at −20 °C.

After thawing, the samples were washed in PT (1 × PBS with Triton X-100) before ultrasonication. For the 0 h to 10 h samples, the cysts were sonicated for three times (3 s each time); 15 h and 20 h samples were sonicated five times; and 40 h–5 d samples were sonicated seven times. Samples from different stages without ultrasonication were used as controls. After washing in PT, the samples were blocked by incubating in 3% H_2_O_2_ for 30 min, and 1 mg/ml BSA for 25 min. The CDH1 primary antibody (Santa, Texas, USA) was added at a 1:50 dilution and the samples were overnight incubated at 4 °C. Controls were incubated in 1 × PBS buffer. All the samples were incubated in HRP-conjugated goat-anti-mouse IgG (Proteintech) antibodies at 37 °C for 1 h, then in SABC (streptavidin biotin complex) for 30 min; washed in PT before being stained with DAB. Finally, the samples were washed and stored in 70% glycerol at −20 °C until microscopic observation.

### Immunofluorescence (IF)

*A. sinica* at different developmental stages (0 h, 15 h and 3 d) were prepared and embedded in paraffin and sliced into 8 μm sections.

The sections were dewaxed and rehydrated before being washed in 0.2% TritionX-100 with PBS for 10 min. Paraffin sections were blocked with 3% BSA at room temperature for 2 h. The rabbit anti-*As*-CDC23 antibody (1:20 in PBST) was added and incubated overnight at 4 °C. Next day the sections were incubated in Cy3-conjugated goat anti-rabbit IgG (diluted 1:50; Proteintech) at 37 °C in the dark for 1 h, and washed with PBST again. Finally, the samples were stored in mounting medium with DAPI (4′, 6-diamidino-2-phenylindole; ZSGB-BIO, Beijing, China) and examined under confocal laser microscopy. The rabbit anti-*As*-CDC20 antibody and mouse anti-CDH1 antibody (Santa) were diluted 1:25 with PBST for double immunofluorescence antibodies, using FITC-conjugated goat anti-rabbit IgG (Proteintech) for CDC20 and TRITC-conjugated goat anti-mouse IgG (Proteintech) for CDH1 as the secondary antibodies.

## Additional Information

**How to cite this article**: Zhang, M. *et al*. APC/C^CDC20^ and APC/C play pivotal roles in the process of embryonic development in *Artemia sinica. Sci. Rep.* **6**, 39047; doi: 10.1038/srep39047 (2016).

**Publisher's note:** Springer Nature remains neutral with regard to jurisdictional claims in published maps and institutional affiliations.

## Supplementary Material

Supplementary Information

## Figures and Tables

**Table 1:** Oligonucleotide primers used in this study.

Primer	Sequence (5′–3′)	Direction
*As-cdc20*F	AGATGGGATTACTGGTGACA	Forward
*As-cdc20*R	TGGAGCGGCAGGTGGTTTCG	Reverse
3′Ou*-As-cdc20*	CCCATCAACATTAGAACAGC	Forward
3′In*-As-cdc20*	ATGCTACGAAACCACCTGCC	Forward
*5*′*As-cdc20*	GCAGGTGGTTTCGTAGCATA	Reverse
RT-*cdc20*F	CTTGCTGTTGGGACGGCT	Forward
RT-*cdc20*R	CGCTGCTAAGAATGTGCT	Reverse
ORF-*cdc20*F	ATGATGAAAAAATCGCAAGAAAAT	Forward
ORF-*cdc20*R	CTATCTGAGTGAAGCGGTGGCGAC	Reverse
*As-cdc23*F	GCTTATCACAGGTATCCAA	Forward
*As-cdc23*R	ACAGGCTCCAGGCATCTCA	Reverse
3′Ou*-As-cdc23*	TACGACTCACTATAGGGCAAG	Forward
3′In*-As-cdc23*	TAGGGCAAGCAGTGGTATCAA	Forward
*5*′*As-cdc23*	GTGTTGAAGGTTCATGTTCTCTGTTGTC	Reverse
RT-*cdc23*F	GCTTATCACAGGTATCCAA	Forward
RT-*cdc23*R	TCCTGAGACACATCTTCCA	Reverse
ORF-*cdc23*F	ATGGAAAAGTCTGACTTAGC	Forward
ORF-*cdc23*R	TCAAGAAATTTTTTTGTATGCA	Reverse
*β*-actinF	AGCGGTTGCCATTTATTGTT	Forward
*β*-actinR	GGTCGTGACTTGACGGACTATAT	Reverse
