# Supplementary material for: APC/CCDC20 and APC/C play pivotal roles in the process of embryonic development in Artemia sinica
Source: Sci Rep. 2016 Dec 19;6:39047. doi: 10.1038/srep39047 (PMC5171921; doi:10.1038/srep39047)

APC/C^{CDC20} and APC/C play pivotal roles in the process of embryonic development in *Artemia sinica*

Mengchen Zhang[a,+], Feng Yao[a,+], Hong Luan[a], Wei Zhao[a], Ting Jing[a], Shuang Zhang[a], Lin Hou[a,*], Xiangyang Zou[b,**]

[a] College of Life Sciences, Liaoning Normal University, Dalian 116081, China

[b] Department of Biology, Dalian Medical University, Dalian 116044, China

[*]Corresponding author:

Lin Hou, College of Life Sciences, Liaoning Normal University, No.1, Liushu South Street, Ganjingzi District, Dalian 116081, China, Tel.: +86 0411 85827082,

Email: houlin@lnnu.edu.cn (L. Hou)

[**]Co-Corresponding author:

Xiangyang Zou, Department of Biology, Dalian Medical University, Dalian 116044, PR China Tel.: +86 0411 86110296;

Email: zouxiangyang@126.com (X. Zou)

[+]These authors contributed equally to this work.

Supplementary Figure Legends

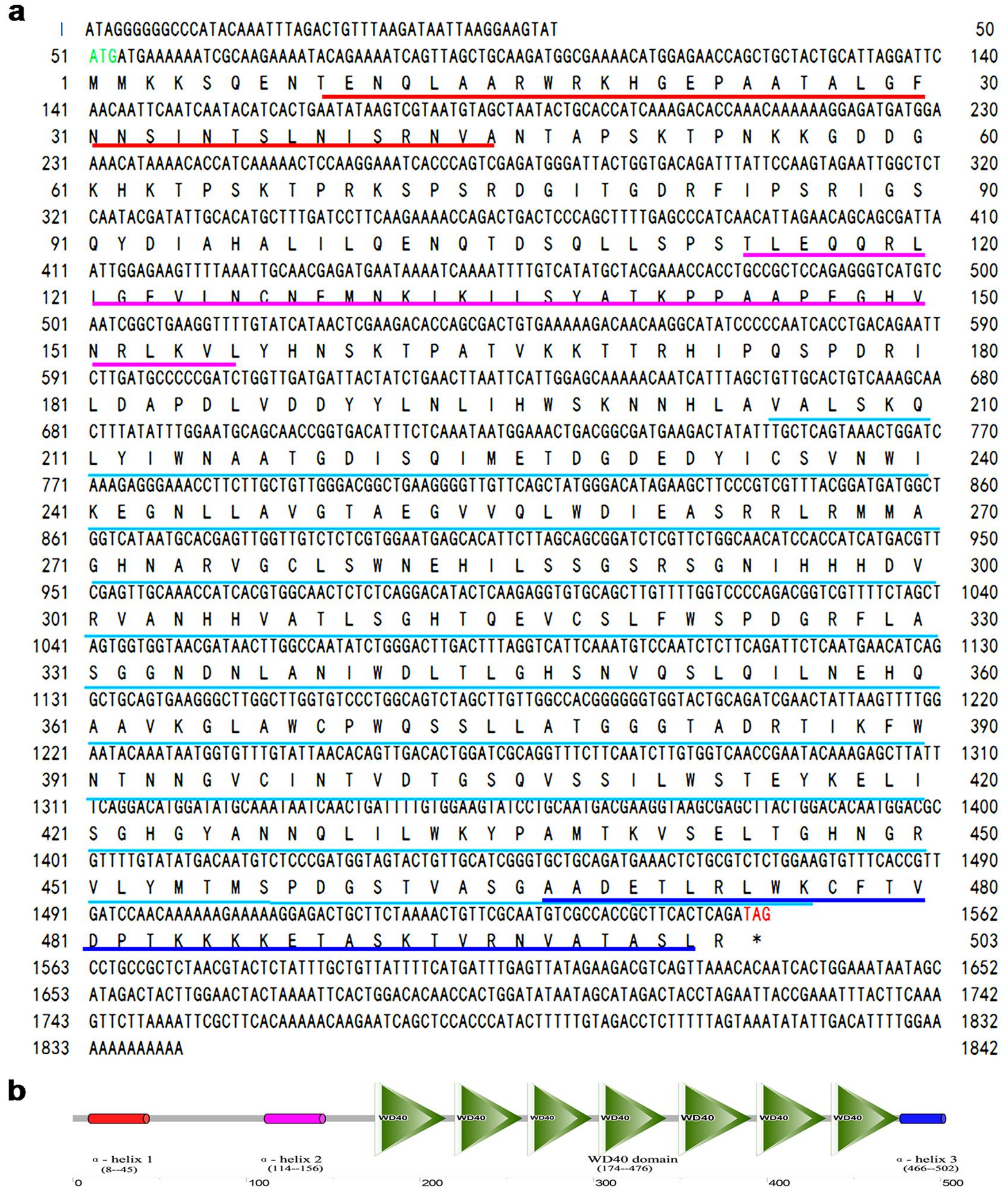

Fig. S1 The full-length cDNA and deduced amino acid sequences of *As-cdc20* and putative protein domains. (A) Sequence analysis of the cDNA and predicted peptide sequences of *As-cdc20*. The start codon is indicated in green; the stop codon is indicated in red; a blue straight line indicates the WD40 superfamily domain. **(B)** Domain analysis of the putative *As*-CDC20 protein, which includes α-helices and seven WD40 repeats.

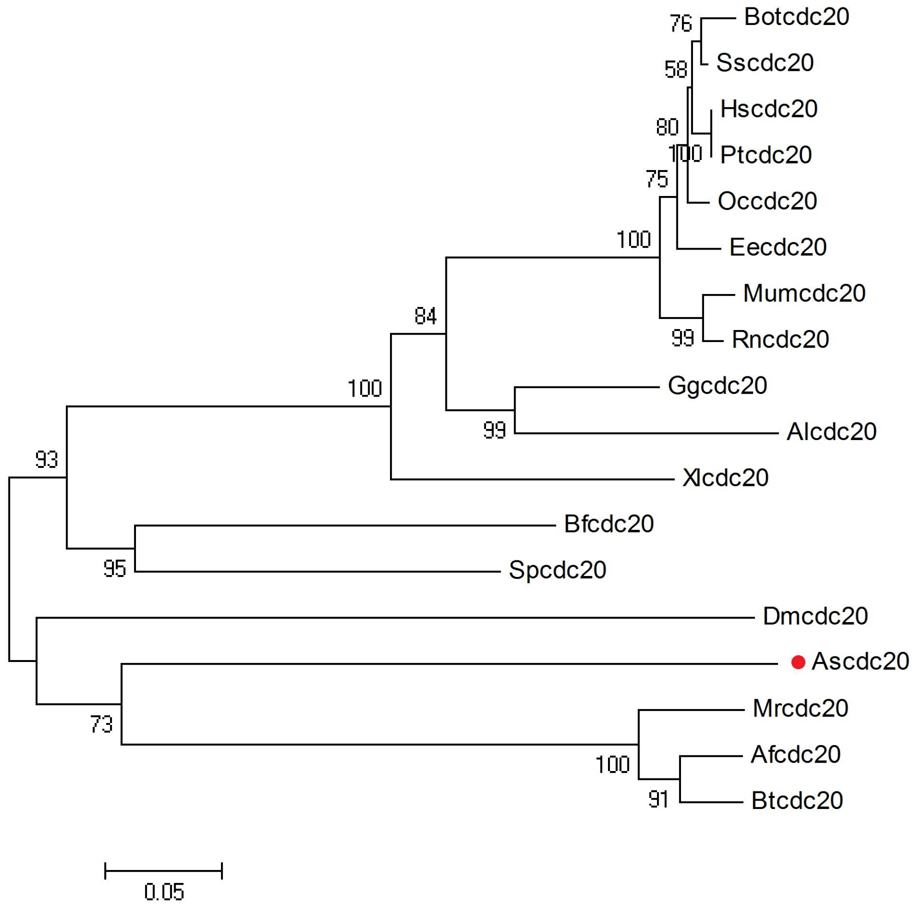

Fig. S2 Multiple sequence alignment of *As*-CDC20 protein. Alignment of known CDC20 sequences from 18 species. Identical amino acid residues are indicated by a black box. Less conserved residues are indicated by a gray box, whereas a pale gray box indicates somewhat similar residues. The sequence of the WD40 domain is shown in red. The sequences and their accession numbers are as follows: Alcdc20, *Alligator sinensis*, XP_006015910.1; Ggcdc20, *Gallus gallus*, NP_001006536.1; Afcdc20, *Apis florea*, XP_003697924.1; Btcdc20, *Bombus terrestri*, XP_003393065.1; Botcdc20, *Bos taurus*, NP_001075905.1; Bfcdc20, *Branchiostoma floridae*, AAO85336.1; Dmcdc20, *Drosophila melanogaster*, AAF53523.1; Eecdc20, *Erinaceus europaeus*, XP_007526147.1; Hscdc20, *Homo sapiens*, NP_001246.2; Mrcdc20, *Megachile*

rotundata, XP_003707890.1; Mumcdc20, *Mus musculus*, NP_075712.2; Occdc20, *Oryctolagus cuniculus*, XP_002715214.1; Ptcdc20, *Pan troglodytes*, JAA40139.1; Rncdc20, *Rattus norvegicus*, NP_741990.1; Spcdc20, *Strongylocentrotus purpuratus*, XP_781780.2; Sscdc20, *Sus scrofa*, NP_001116566.1; Xlcdc20, *Xenopus laevis*, NP_001081776.1; Ascdc20, *Artemia sinica*, KP162166.

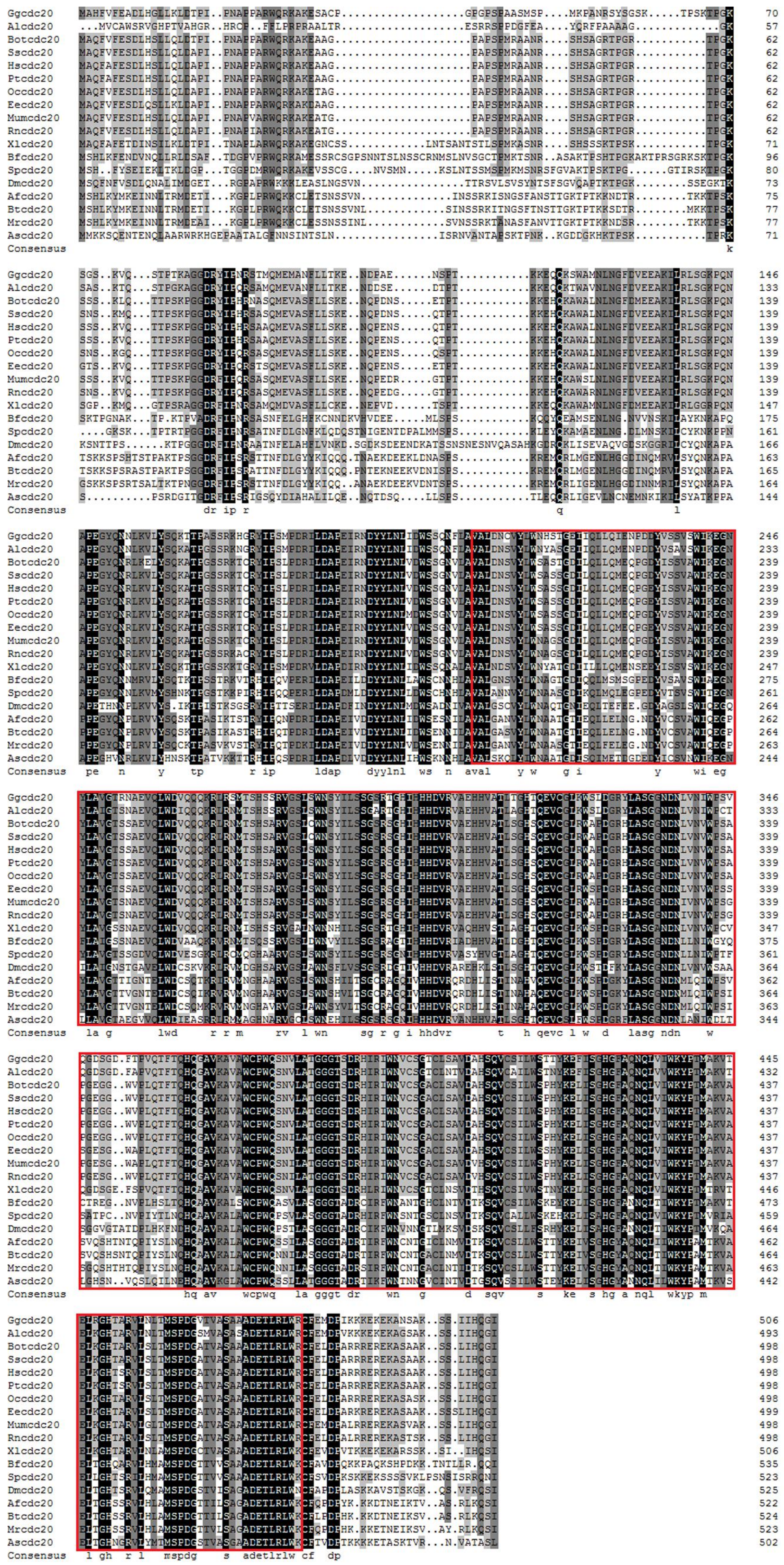

Fig. S3 The neighbor-joining phylogenetic analysis of *As*-CDC20 protein. The tree constructed based on the amino acid sequences of *As*-CDC20 (this study) and 17 other

species from GenBank using the sequence analysis tool MEGA 4.1. The sequences and their accession numbers refer to the legend of Supplementary Fig. S2. A red dot indicates *As*-CDC20.

a

```
1      gatttgaaatattctaaagATGGAAAAGTCTGACTTAGCTCATGAGCTTATCACAGGTAT
1                         M  E  K  S  D  L  A  H  E  L  I  T  G  I
61     CCAAGCTTGTCAAGATCGAGGGTTACTTCATTCTAGCAAGTGGCTCTCTGAATTATTGTA
15      Q  A  C  Q  D  R  G  L  L  H  S  S  K  W  L  S  E  L  L  Y
121    TGCCCTTGATGATAAAAATACCAACTGTATCCCTACACCTCAAAAATGCATTTTGAACGA
35      A  L  D  D  K  N  T  N  C  I  P  T  P  Q  K  C  I  L  N  E
181    AACATCTGAAGTGCAGATGGAAGATGTGTCTCAGGATAGAACATTTATTCTTTCAAAGTG
55      T  S  E  V  Q  M  E  D  V  S  Q  D  R  T  F  I  L  S  K  C
241    CTACTTTGACCTCAAGGAATATGACAGGTGTGCATATTTTTCTAAAAAACTGAAGAGCAA
75      Y  F  D  L  K  E  Y  D  R  C  A  Y  F  S  K  K  L  K  S  K
301    AAAAGGAAAGTTTTTATATTGGTACTCACAATATTTATCAGGGGAAAAGAAGAGGATTGA
95      K  G  K  F  L  Y  W  Y  S  Q  Y  L  S  G  E  K  K  R  I  E
361    AGATATGTCAGAGTCAGAAATGACAACAGAGAACATGAACCTTCAACACTTGACTGAATT
115     D  M  S  E  S  E  M  T  T  E  N  M  N  L  Q  H  L  T  E  L
421    AAAGCAAGAATTAAGTGTTGCATACAAAAAAATTTCTTGAgatgcctggagcctgttagg
135     K  Q  E  L  S  V  A  Y  K  K  I  S  *
481    gcaagcagtggtatcaatccgagtttgcatctactatcgagtgatattgtataggatttg
541    ctcaagcaaaagagaaagcaagtgctgaaggagggaccgttgaaccagaaaataaaaaac
601    aagcagcatccaaaatggaaagacaacaagtacccgtaatggaaggagagatgtatgatg
661    aaagaagagatcccgaattcaacccagctagtgaaagggaattcattgaaacggaagaac
721    cgtctcgcaaccctaaacaagaggaggaagaagccaatagagaaattcaagaacaaagcc
781    gtcaaatggggatcgacccaagtaaaactggtcatgaaactcgaactggtctagactatc
841    cgactagtcaggatcgagtcactaaacacaaagaggagtatattgaaaaaggagaggaag
901    acattccttcggttgagtctggagttggaaagaatcctgagcaggtggccaaaggaagaa
961    aagcggctgctggtactactcacgaagagttcgttgagcgtgggaaaaaaggggctgaag
1021   cccgatggggaaaacagcaaggatctcttgctcgaaatcaataaccctctgagaacagaa
1081   aactaattaccttttttgagttatttgacttcgatttcttaatatagcttaatgtagctg
1141   atcaaaatgttgcacctgtgcagattaaaactttttcatttcttcgaaaaaaaaaaaaa
1201   aaaaaaaaaaaaaaa
```

b

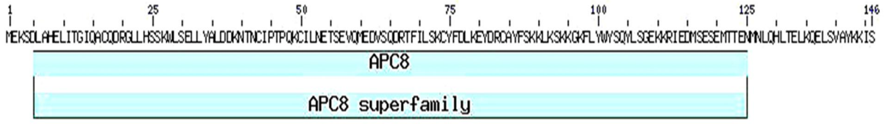

Fig. S4 The full-length cDNA and deduced amino acid sequences of *As-cdc23* and putative protein domains. (A) Sequence analysis of the cDNA and predicted peptide

sequences of *As-cdc23*. A black straight line indicates the ORF region. **(B)** Domain analysis of the putative *As*-CDC23 protein. The mature protein includes an APC8 superfamily domain in the N-terminus.

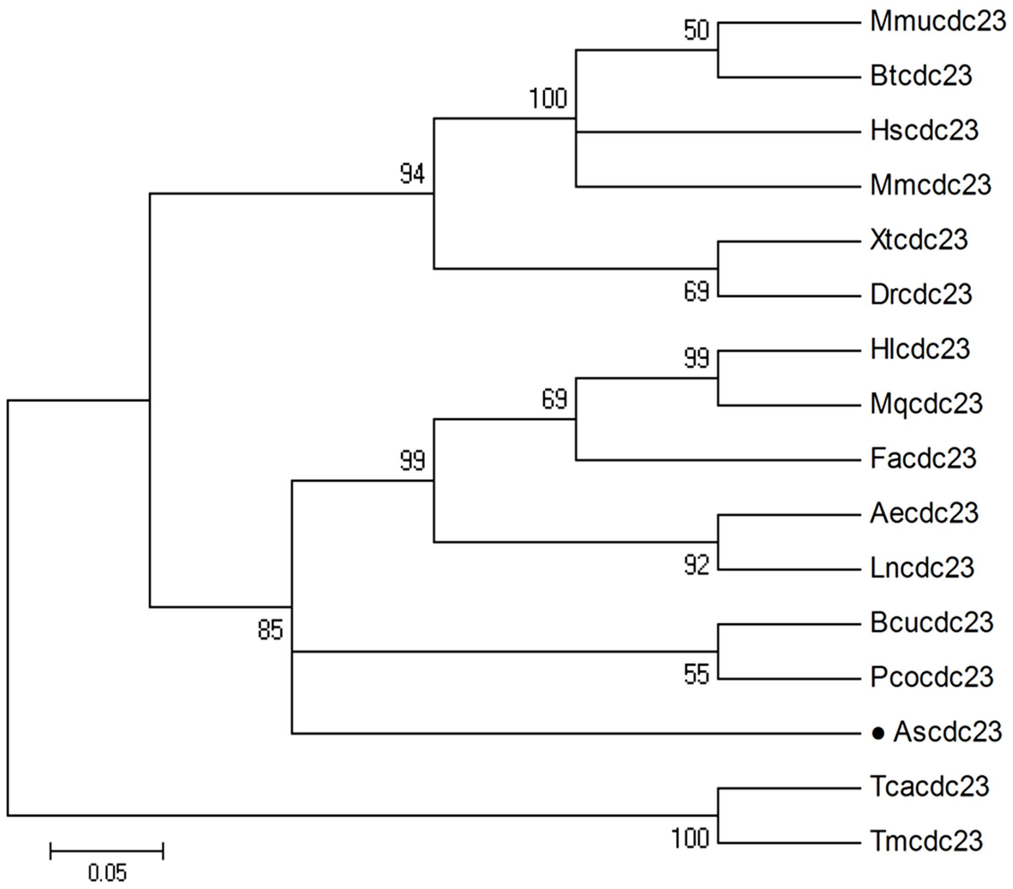

Fig. S5 Multiple sequence alignment of *As*-CDC23 protein. Sequence alignment of known CDC23 sequences from 16 species. Identical amino acid residues are indicated by gray shading, and less conserved residues are indicated by pale gray shading. The sequence of the APC8 domain is shown in blue. The sequences and their accession numbers are as follows: Mmucdc23, *Mus musculus*, NP_848124.1; Btcdc23, *Bos taurus*, NP_001073735.1; Hscdc23, *Homo sapiens*, NP_004652.2; Mmcdc23, *Macaca mulatta*,

NP_001244486.1; Xtcdc23, *Xenopus tropicalis*, NP_001016759.1; Drcdc23, *Danio rerio*, NP_957227.1; Hlcdc23, *Habropoda laboriosa*, KOC68110.1; Mqcdc23, *Melipona quadrifasciata*, KOX74256.1; Facdc23, *Fopius arisanus*, JAG81156.1; Aecdc23, *Acromyrmex echinatior*, EGI60542.1; Lncdc23, *Lasius niger*, KMQ88026.1; Bcucdc23, *Bactrocera cucurbitae*, JAC97421.1; Pcocdc23, *Pediculus corporis*, XP_002426290.1; Tcacdc23, *Toxocara canis*, KHN78208.1; Tmcdc23, *Trichinella murrelli*, KRX39430.1; Ascdc23, *Artemia sinica*, KU986660.

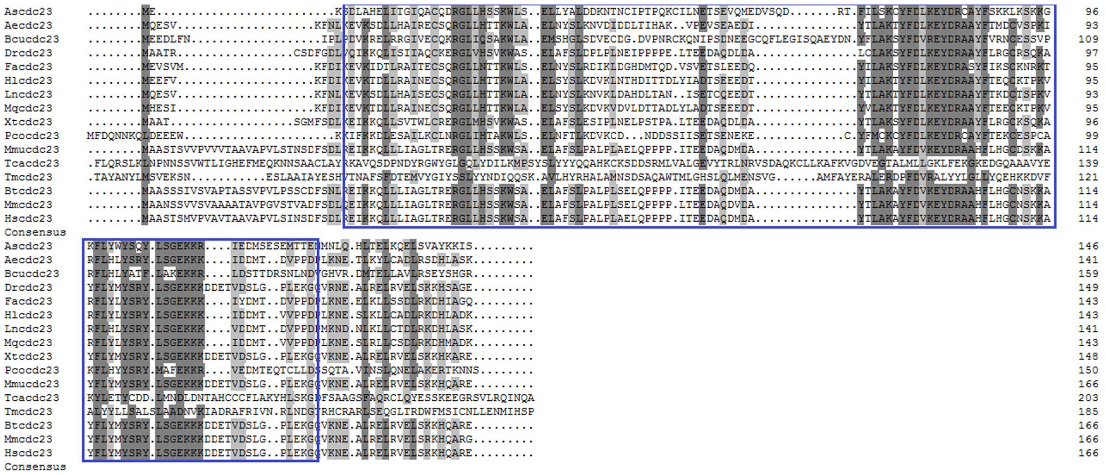

Fig. S6 The neighbor-joining phylogenetic analysis of *As*-CDC23 protein. The tree constructed by the neighbor-joining method, based on the amino acid sequences of *As*-CDC23 and 15 other species from GenBank using the sequence analysis tool MEGA 4.1. The sequences and their accession numbers are shown in Supplementary Fig S5. A black dot (●) indicates *As*-CDC23.

Equipment and settings

Fig.S1 and Fig.S4: We used DNAMAN to display the sequences, and using ORF Finder; the prosite tools of ExPASy and SMART predict the protein structural and functional domains.

Fig.S2 and Fig.S5: MEGA4.1 software and ClustalX2.0 were used to construct a phylogenetic tree of the two proteins from different species using the neighbor-joining (NJ) method.

Fig.S3 and Fig.S6: Multiple sequence alignments were performed for the amino acid sequences of *As*-CDC20 and *As*-CDC23 with similar proteins from other species, using the ClustalX2.0 and DNAMAN.

Supplementary Figure 7: The full-length gels of *As*-CDC20 protein.

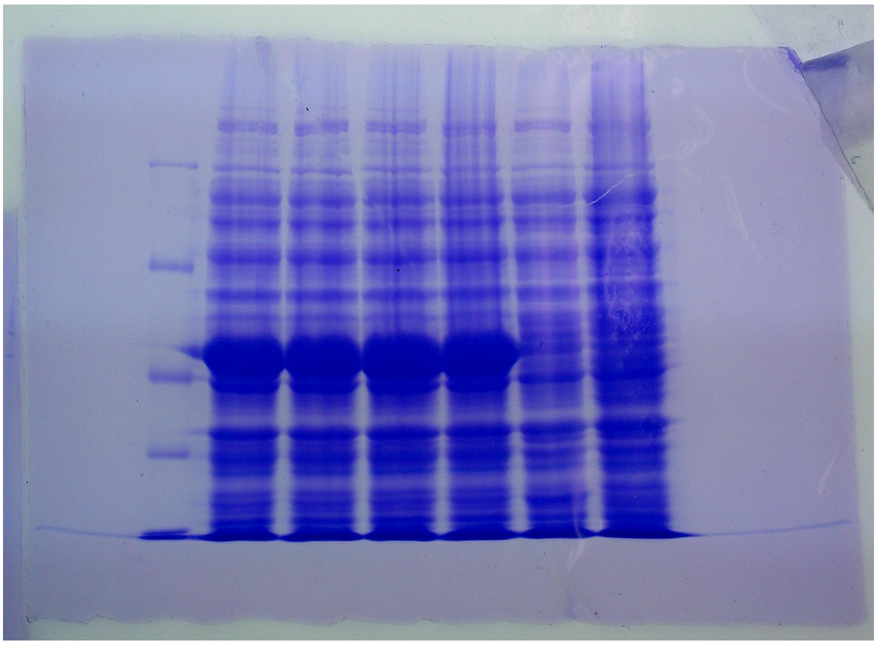

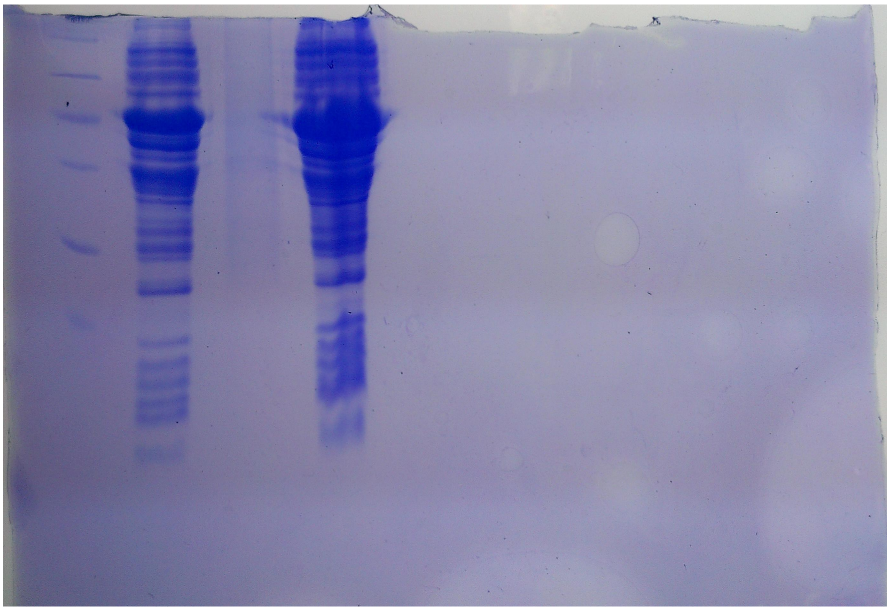

Supplementary Figure 8: The full-length gels of *As*-CDC23 protein.

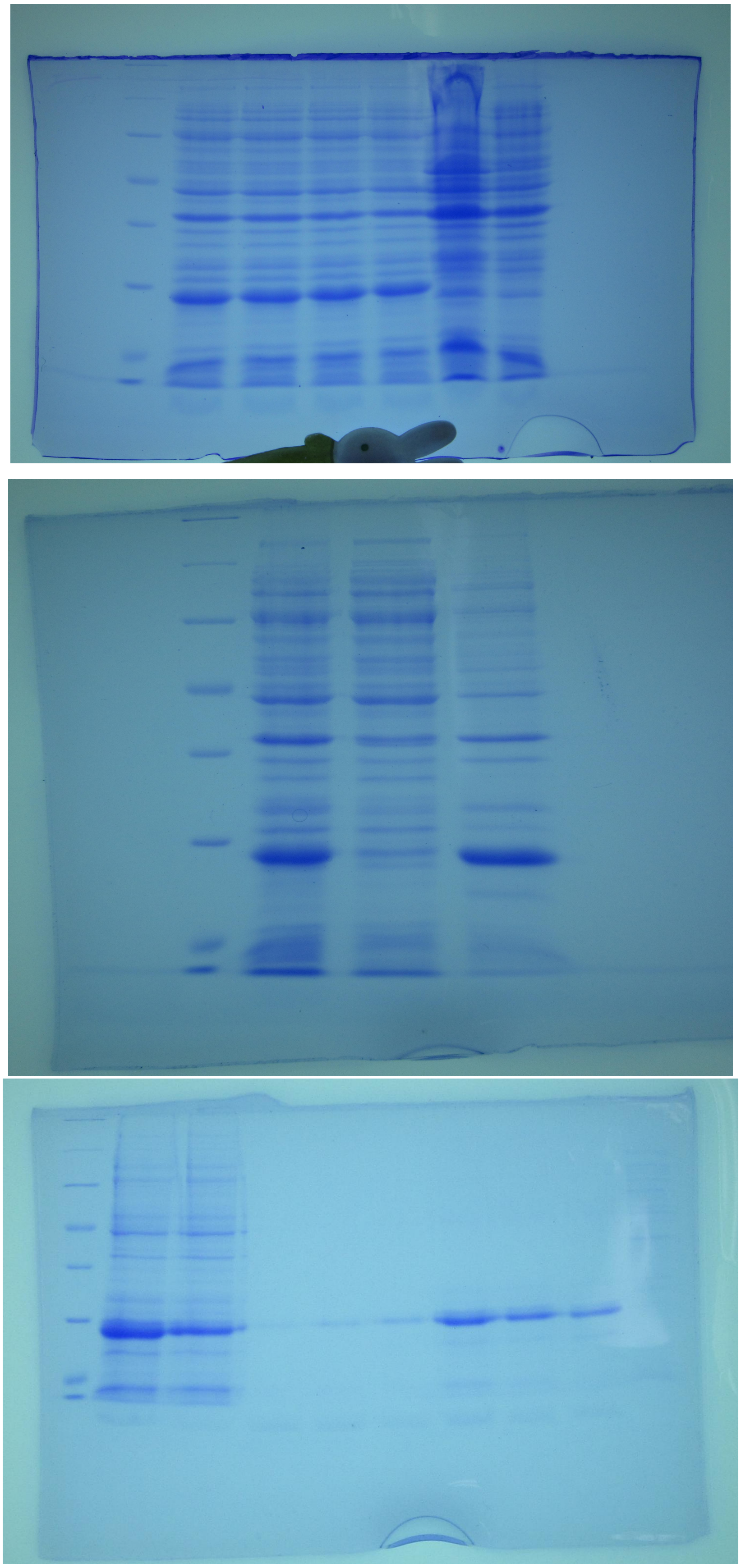

Supplementary Figure 9: The full-length blots of western blotting analysis.

GAPDH

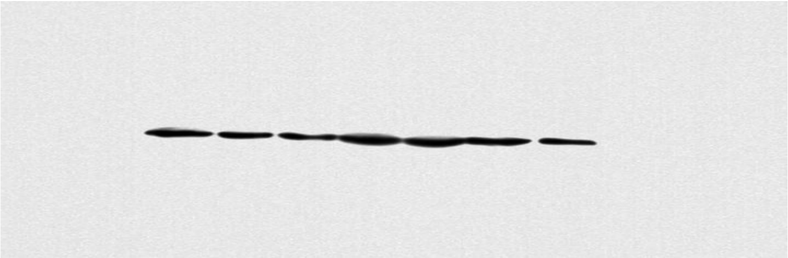

***As*-CDC20**

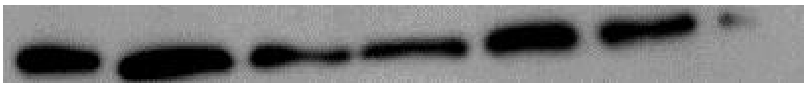

***As*-CDH1**

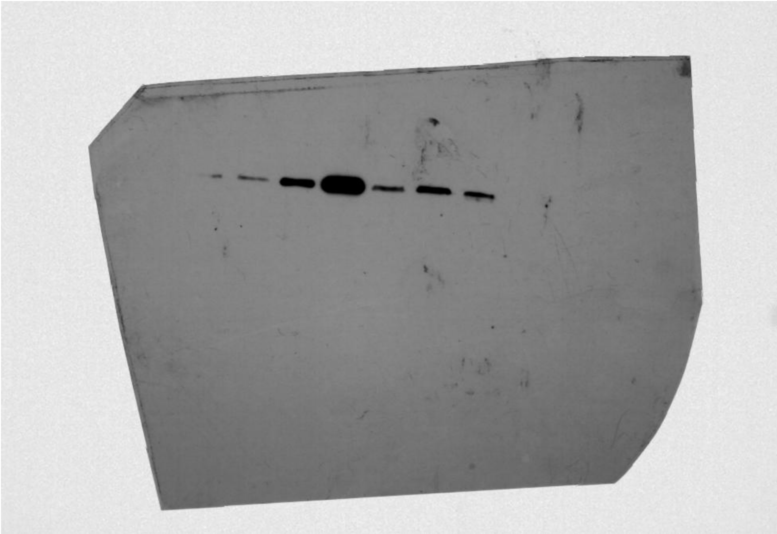

***As*-CDC23**

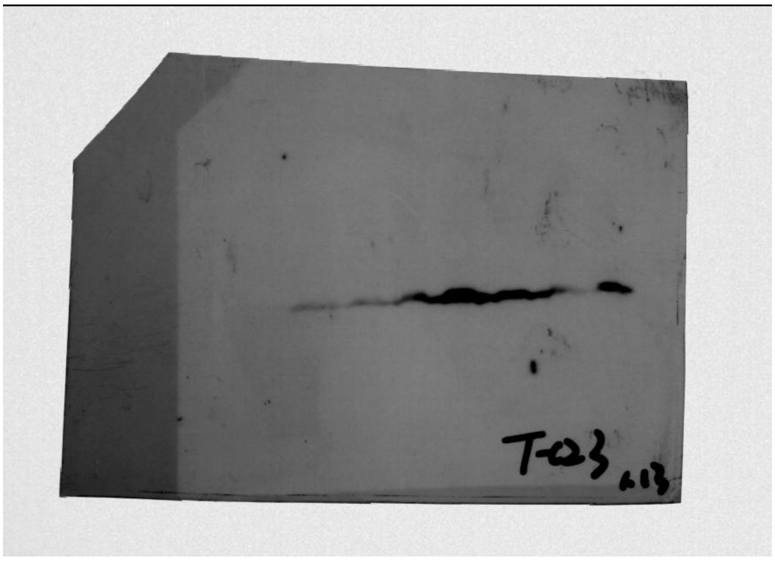

***As*-Geminin**

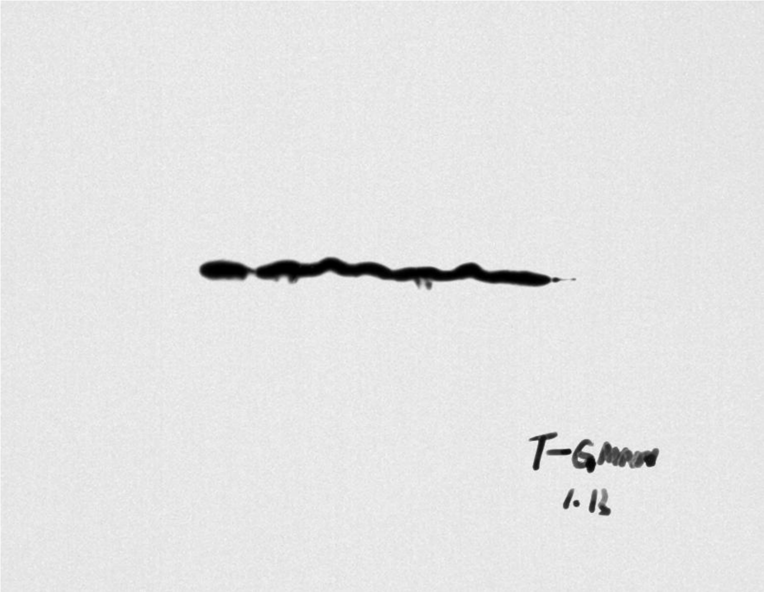

***As*-CDC14**

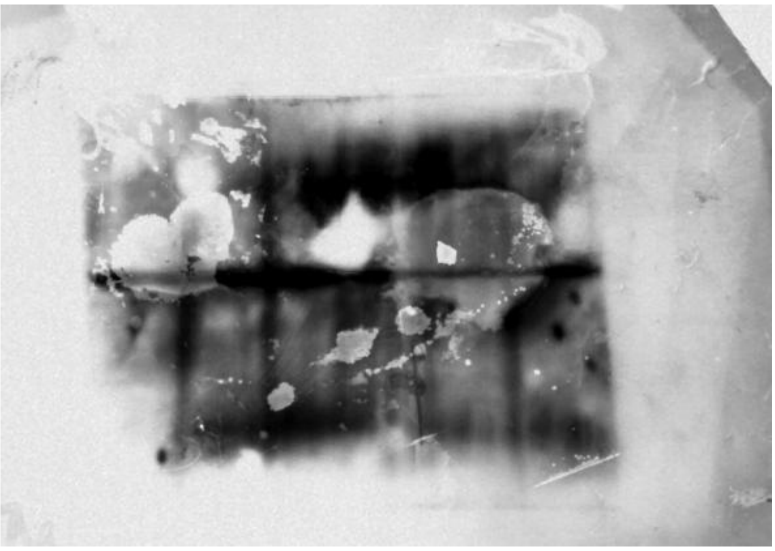

***As*-securin**

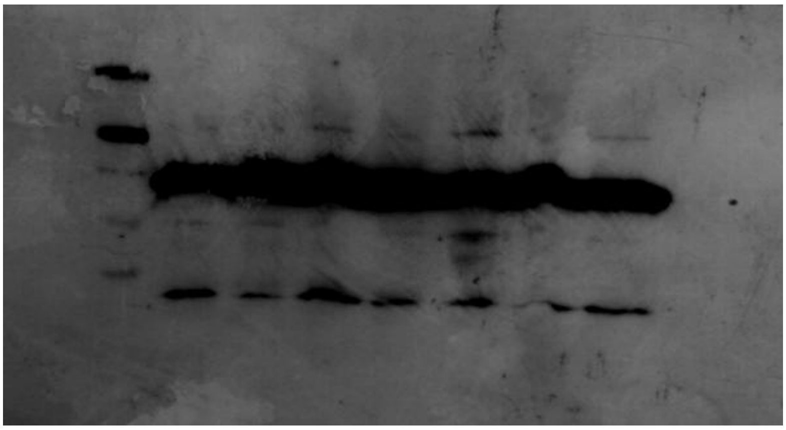

***As*-CDK1**

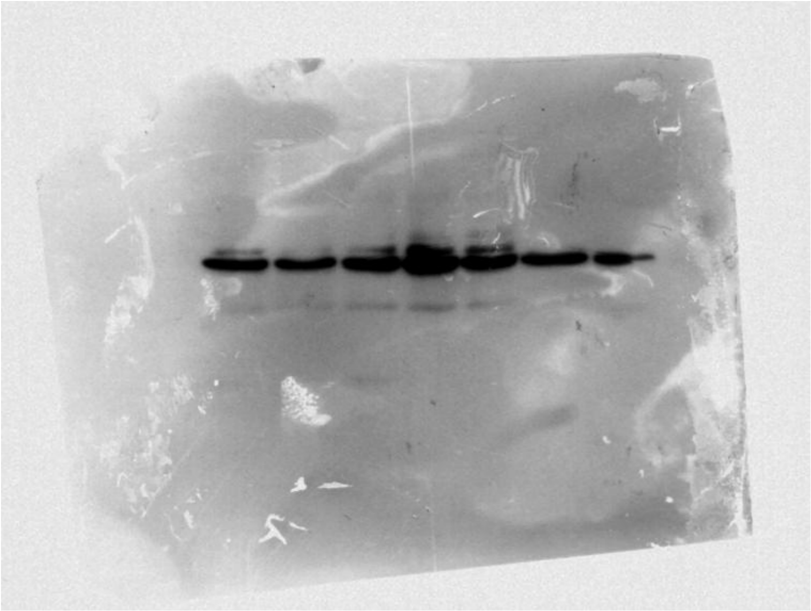

***As*-Cyclin B**

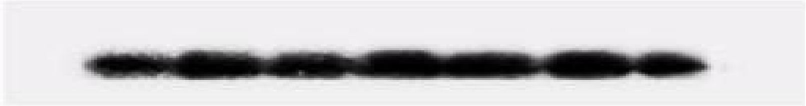

Supplement: Supplementary Information [file srep39047-s1.pdf]
